# Mandibular Cortical Bone Evaluation in a Healthy Paediatric Population

**DOI:** 10.3390/healthcare11243105

**Published:** 2023-12-05

**Authors:** Marilena Kotsanti, Anastasia Mitsea, Nikolaos Christoloukas, Kyriaki Seremidi, Sotiria Gizani

**Affiliations:** 1 Department of Paediatric Dentistry, School of Dentistry, National and Kapodistrian University of Athens, 115 27 Athens, Greece; 2 Department of Oral Diagnosis and Radiology, School of Dentistry, National and Kapodistrian University of Athens, 115 27 Athens, Greece

**Keywords:** cortical bone, quality, quantity, children, adolescents, dental panoramic radiographs

## Abstract

Background: Changes in mandibular cortical bone have been documented in children with chronic diseases but not in healthy populations. Aim: We aimed to qualitatively and quantitatively evaluate the mandibular cortical bone of a healthy paediatric population using dental panoramic radiographs (DPTs). The secondary objective was to assess the effect of dental status on cortical bone. Design: The DPTs of 660 individuals, aged 6–18 years, were evaluated by two blinded examiners. Bone was qualitatively categorized according to the Mandibular Cortical Index (MCI), and its width was calculated using the Mandibular Cortical Width Index (MCW). Differences between gender and age were tested using Fisher’s exact and correlation with dental status with the chi-square test. Results: A significant correlation was observed between bone morphology and gender, with girls having more frequent even and sharp endosteal margins than boys, who showed semilunar defects. The degree of erosion increased with age, as did bone thickness. A positive correlation between thickness and type of dentition was recorded, with normal bone predominating in children in the mixed dentition stage. Νο correlation was found between bone morphology or thickness and the factors associated with dental status. Conclusions: Bone quality and quantity vary significantly between different genders and age groups. Dental needs and treatment characteristics did not seem to affect the above parameters.

## 1. Introduction

Bone development is a complex process involving interrelated events in space and time. Bone mineral density (BMD) increases during childhood and adolescence until peak bone mass is attained. These periods are critical as high skeletal growth of up to 90% of adult bone mass is acquired [1]. Thus, it is necessary to define normal values for bone density during this period to allow for the determination of deviations. Early detection and adequate treatment are essential for the prevention of osteoporosis and fractures later in life.

Assessing bone health in paediatric populations necessitates the utilization of reliable and sensitive diagnostic tools and techniques. Radiographic imaging, such as dual-energy X-ray absorptiometry (DXA), remains the gold standard for evaluating bone mineral density. However, emerging methodologies, such as quantitative ultrasound (QUS) and peripheral quantitative computed tomography (pQCT), offer additional means of assessing bone quality, microarchitecture, and biomechanical properties [2].

Since more than 1.5 billion dental radiographs are obtained annually [3], these radiographs may be useful in detecting general conditions that have early and distinct manifestations in the jawbones along with diagnosing dental problems. Furthermore, these radiographs may contribute to the identification of problems in the jawbones related to the treatment of systematic diseases.

The application of radiomorphometric indices in panoramic radiographs, as an indicator of alterations in BMD, has been used to screen and identify adults at risk of osteoporosis [4,5]. Based on the results of the 3-year OSTEODENT project, a 3 mm limit for the cortical width in the mental foramen region was established, and patients with a smaller cortical width should be referred for definitive diagnosis [6]. Relative studies in children are rare and focus mainly on changes in bone mass density in pathological conditions. Panoramic radiographs have been used in studies to record bone density in children with conditions that impair growth, such as preterm births [7], osteogenesis imperfecta [8], HIV infection, and cancer [9,10]. Additionally, Yasa et al.’s study [11] on the impact of obesity on the mandibular cortical bone revealed that individuals who are obese and overweight have bigger mandibular cortexes than those who are of normal weight.

In recent years, there has been growing concern regarding the prevalence of bone-related disorders among children and adolescents. Factors such as sedentary lifestyles, poor nutrition, and the increasing prevalence of obesity have contributed to a higher incidence of bone-related issues, including fractures, osteoporosis, and reduced bone mineral density [12]. Consequently, there is a critical need to investigate bone health in an otherwise healthy paediatric population to identify potential risk factors, implement preventive measures, and optimize interventions. Developing reference tables for bone quality and quantity is important as it can lead to the early detection of low BMD, which is linked to a variety of metabolic disorders. Children’s bone growth may be negatively impacted by primary bone abnormalities including osteogenesis imperfecta, as well as illnesses that cause chronic inflammation, malabsorption, immobilization, haematological disorders, delayed sexual maturity, or gonadal insufficiency. Acute lymphocytic leukaemia, Crohn’s diseases, cystic fibrosis, cerebral palsy, thalassemia, cerebral palsy, and anorexia nervosa are a few examples of these. The development of new bones is also threated by medical treatments like glucocorticoids. The main aim of this study was to thoroughly document and evaluate, qualitatively and quantitatively, the mandibular cortical bone of a healthy paediatric population, aged 6–18 years, using dental panoramic imaging. Secondary objectives included assessment of the distribution of bone quality and density in different gender and age groups and the effect of factors that can potentially influence occlusion and indirectly masticatory stresses on the above variables.

Therefore, the null hypothesis tested was that cortical bone quality and quantity are normally distributed between genders and different age groups and not affected by any factors related to the stability of the occlusion.

## 2. Material and Methods

This double-blinded retrospective cohort study complied with the ethical principles outlined in the Helsinki Declaration [13]. The research protocol was submitted to and approved by our University’s Ethics Committee School of Dentistry, National and Kapodistrian University of Athens, Greece.

### 2.1. Sample Selection

The archives of the School of Dentistry, National and Kapodistrian University of Athens, Greece were searched to identify patients aged 6 to 18 years with updated dental and medical records that had at least one dental panoramic radiograph of good quality taken in the context of their dental treatment. The radiographs should have been taken between 2012 and 2021, a period that ensured comparability since they had been performed with the same panoramic equipment (Planmeca ProMax Elsinki Finland 50/60 Hz, 70 kV at 8 mA for 15 s), and the possible distortion was therefore constant.

Exclusion criteria were panoramic radiographs of:

- Patients with chronic diseases/conditions/treatments affecting bone, e.g., eating disorders, musculoskeletal disorders, etc.;
- Patients born prematurely or during early puberty;
- Patients undergoing/having undergone orthodontic treatment;
- Poor quality.

From all eligible patients, the ones included in the study were randomly selected by a third person not involved in the evaluation process by generating random numbers using a computer-based system.

### 2.2. Sample Size

A power analysis with a strength of 0.8 was carried out. The power analysis aimed at a target power (1 − β) of 0.8, signifying an 80% likelihood of detecting a genuine effect [14] (Bartlett, 2022). Utilizing the Power Size Analysis module in STATISTICA for Windows 12.5, sample size estimation was conducted to achieve a well-distributed patient population across age and gender categories, ensuring the study’s robustness and generalizability. Specifically, the determined sample size was 660 patients. To attain this, participants were stratified into gender groups of equal size (*n* = 15) and age groups with a 2-year granularity (*n* = 30 per year, totalling *n* = 60 per age group).

It is crucial to highlight that the selection of a power level of 0.8 adheres to the standard in statistical analysis, representing a balanced trade-off between the risks of type I and type II errors. In simpler terms, power is the probability of avoiding a type II error (1 − β), and conventionally, a threshold of 0.8 (80%) is considered acceptable, although different thresholds may be appropriate based on the study design [15] (Serdar et al., 2021).

The parameters guiding the calculation align with established statistical principles and conform to widely accepted practices in sample size determination. There are four primary variables influencing power—alpha (α), sample size, effect size, and variance [16] (Madjarova et al., 2022). The predetermined value of α was a priori set at 0.05, serving as the threshold for accepting or rejecting the null hypothesis. Modifying the sample size is another means of adjusting power, but practical constraints and economic considerations may limit the feasibility of increasing sample size, potentially causing project delays. Consequently, the determination of sample size involves a delicate balance that hinges on the variables under study (in our case, a continuous variable) and the study design, whether it is within-subjects or between-subjects [17] (Uttley, 2019).

### 2.3. Study Procedure

Initially, a paediatric dentist who was trained and calibrated mixed and prepared the radiographs for the analysis, including drawing the lines required for the evaluation and cropping the radiographs so that only the lower jaw was visible to assist the analysis. The same observer collected the data regarding the dental status of the patients at the time the panoramic radiograph was taken. Information was obtained about the status of the dentition, the number of missing teeth due to age and caries, and teeth with extensive resin composite restorations and stainless-steel crowns.

Another two calibrated dentists specialized in oral radiology (and blinded to the patient’s demographic characteristics) applied qualitative and quantitative anthropometric indices to all radiographs in random order. Any disagreements between observers were addressed through discussion, and if an agreement could not be reached, a third evaluator not previously involved in the foregoing processes was consulted. Both observers re-examined 100 randomly selected radiographs after one month to assess intra- and inter-observer reliability.

### 2.4. Qualitative Bone Assessment (Mandibular Cortical Index, MCI)

Observers classified the morphologic changes in the mandibular cortex on each panoramic radiograph using the three-stage scale developed by Klemetti et al. [18]:

C1: the endosteal border of the cortex is even and sharp bilaterally;

C2: the endosteal border demonstrates semilunar deficiencies (lacunar resorption) and/or seems to create endosteal cortical residues unilaterally or bilaterally;

C3: the cortical layer forms substantial endosteal cortical residues and is clearly porous.

In each panoramic radiograph, the mandibular cortex, distal to the mental foramen, was identified and assessed bilaterally. The side with the most severe stage was recorded for each dental radiograph.

### 2.5. Quantitative Bone Assessment (Mandibular Cortical Width, MCW)

**Figure 1:**
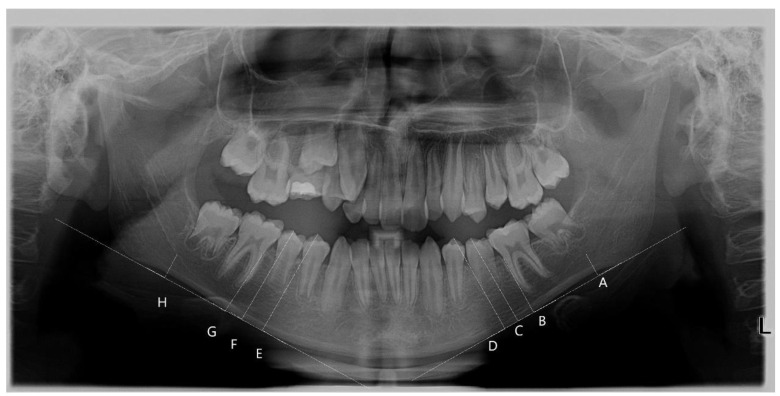
Panoramic radiograph showing the sites (A–H) where measurements of the width of the cortical bone were performed. Lines were drawn perpendicular to a tangent at the lower border of the cortex to the inner border of the cortex at A and H (the deepest points of the antegonial notch concavity), at the cementoenamel junction of the crown of the first molar (B, G) and at the highest cusp of the premolars (C–F). The width of the cortex was measured along these lines.

Observers evaluated the thickness of the mandibular cortical bone bilaterally, according to the modified index [7] adopted for paediatric populations. A line was drawn along the bottom border of the mandible on each side, followed by four perpendicular lines to the tangent at the positions shown in Figure 1:

1. Antegonion—the deepest point of the antegonial notch concavity (A, H);
2. The mesial cementoenamel junction of the first molar perpendicular to the mandibular base (B, G);
3. The most superior cusp tip of the second premolar perpendicular to the mandibular base (C, F);
4. The most superior cusp tip of the first premolar perpendicular to the mandibular base (D, E).

Image J (Image J 1.50c4 for Windows XP) was used to quantify the width of the cortical bone where the vertical lines came into contact with it. The mean value from the two measurements for each site, as well as the overall mean value for all sites, was calculated and recorded for each radiograph. Pixel values (1024 × 1024 pixels; 8-bit; 1 MB) recorded by the software were converted into mm using a calculated coefficient factor from a sample of known diameter [10].

### 2.6. Statistical Analysis

Mean values for the cortical bone thickness at each site were calculated, and age and gender distribution are presented. The prevalence of bone morphology (left, right, and total) was calculated for each age group and gender. To estimate the correlation between bone morphology and demographic characteristics, Fisher’s exact test and z-test for comparing two proportions were applied. The agreement of calculated values of cortical bone width between the observers was assessed by applying Lin’s concordance correlation coefficient measure, Pearson’s r correlation coefficient, and calculating the 95% Limits of Agreement (LoAs). Lin’s concordance correlation coefficient ranges from 0 to 1, and the closer the value is to 1, the more statistically significant the correlation is. The correlation between dentition phase and dental status and demographic characteristics and bone morphology was evaluated by applying the chi-square test (or, in the case that the assumptions did not hold, Fisher’s exact test). Spearman’s rho correlation coefficient was calculated to assess the correlation of the sample characteristics by age (years), and one-way analysis of variance (ANOVA) was conducted to assess differences in the sample characteristics by mean age and by total cortical bone thickness, followed by Bonferroni correction for multiple comparisons. STATA 13 was the software employed for all analyses (StataCorp. 2013. Stata Statistical Software: Release 13. College Station, TX, USA: StataCorp LP). Two-tailed *p*-values are reported. A *p*-value of less than 0.05 was considered statistically significant.

## 3. Results

### 3.1. Demographic Characteristics

The sample consisted of 326 males (49%) and 334 females (51%), with a mean chronological age of 11.7 years (SD 3.37). All in all, 31% percent of the patients were in the early mixed dentition phrase, 23% in the late mixed phrase, and 46% in the permanent dentition phrase. The majority of patients (90%) did not have caries, composite resin restorations (79.2%), or SCC (95.2%). Ten percent had at least one missing tooth. The distribution of the above variables did not differ statistically significantly between boys and girls (*p*-value > 0.05). A statistically significant negative correlation was observed between age and the presence of caries, SCC, and missing teeth (*p*-value < 0.05). Finally, a statistically significant difference in mean age was observed in dentition (*p*-value < 0.001). Mean age differed significantly between all possible dentition pairs (Bonferroni *p*-value < 0.001).

### 3.2. Observer Reliability

Both intra- and inter-observer reliability were >0.87 for qualitative cortical bone assessment. The mean difference in cortical bone thickness values between Observer 1 and Observer 2 was very small (0.17 with SD: 0.15), while the 95% LoAs were estimated to range from −0.13 to 0.46. Lin’s concordance correlation coefficient was 0.90, indicating excellent agreement of calculated values between the two observers. Moreover, Pearson’s correlation coefficient presented an excellent linear correlation (0.96) with both measures that was statistically significant (*p*-value < 0.001).

### 3.3. Qualitative Cortical Bone Assessment

The mandibular cortex’s endosteal edge was even and sharp on both sides in 80% of the subjects (88% right and 85% left), while 19% of the subjects displayed semilunar deficiencies (lacunar resorption) and/or seemed to have generated endosteal cortical residues on one side (12% right and 15% left) or bilaterally. There was one patient that had a clearly porous cortical layer with heavy endosteal residues. The distribution of morphology did not differ between the left and right side in a statistically significant way (*p*-value > 0.05).

Table 1 presents the prevalence of bone morphology (bilaterally) according to gender, indicating a statistically significant correlation (*p*-value = 0.026). Specifically, an even and sharp endosteal margin was more frequent in girls compared to boys (84.1% vs. 76.9%). The endosteal margin presented semilunar defects (lacunar resorption) and/or seemed to form endosteal cortical residues on one or both sides more frequently in boys 22.8% than in girls 15.9%.

A similar significant correlation (*p* = 0.013) was observed in the distribution of bone morphology by age (Table 2). The endosteal margin of the cortex was even and sharp on both sides more frequently in patients aged 8 to 11 years (86.6% and 88.5%, respectively) compared to other age groups. On the other hand, the endosteal margin showed semilunar defects (lacunar resorption) and/or seemed to form endosteal cortical residues on one or both sides more frequently in patients aged 6 to 7 years and >14 years (33% for both groups).

Bone morphology in each age group, stratified by gender, showed a significant correlation in boys only (*p*-value < 0.001), showing that an even and sharp cortex on both sides was more frequent in patients aged 8 to 11 years (90.1% and 92.6%) compared to other ages.

### 3.4. Quantitative Cortical Bone Assessment

**Figure 2:**
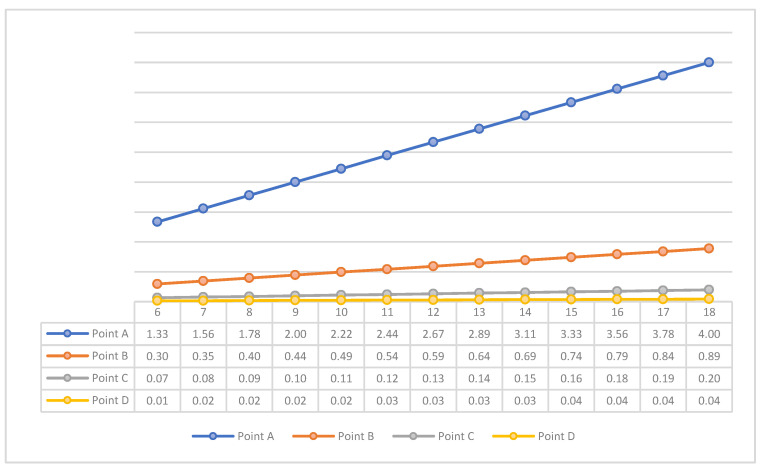
Distribution of mean cortical width on each point according to age.

A statistically significant positive relationship was found between all points assessing bone thickness and age (years), meaning that as age increases, bone thickness increases accordingly (*p* < 0.001) (Figure 2). Multiple comparisons between total bone thickness and age groups resulted in statistically significantly lower mean values in children ages 8–9 compared to children ages 14–15 years (mean difference: −0.9449, *p*-value = 0.010) and children ages 18 years (mean difference: −1.1412, *p*-value = 0.046).

Distribution of mean bone thickness by age group and by gender showed a statistically significant difference in both boys and girls. Specifically, boys had a statistically significantly lower mean value at ages 8–9 years compared to children aged 18 years (mean difference: −1.6525, *p*-value = 0.026). Regarding girls, a statistically significantly lower mean value of total bone thickness was found in ages 6–7 and 8–9 years compared to ages 16–17 years (mean difference: −0.1116, *p*-value *= 0.040* and −0.2374, *p*-value *= 0.016*, respectively).

### 3.5. Correlation of Cortical Bone with Dental Status

Table 3 summarizes the correlation between bone morphology and the samples’ dental status. The statistical tests using bone morphology according to the total MCI was applied, and in order to have sufficient frequency in all categories to perform the statistical test, only C1 and C2 were evaluated. Samples’ dental status and stage of dentition were not statistically significantly associated with bone morphology according to the total MCI (*p*-value > 0.05).

Table 4 presents the association between sample characteristics and total mandibular cortical bone thickness value. The differences were not statistically significant (*p*-value> 0.05), except for the stage of dentition (*p*-value < 0.001), where the mean value of cortical bone thickness was significantly higher in children in permanent dentition compared to those in the early and late mixed dentition stages (*p*-value < *0.001* and *p*-value = *0.049*, respectively).

Table 5 and Table 6 display the mean normal values for bone quality and quantity by gender for each age group, which can be used as reference charts for cortical bone morphology and thickness in a healthy Greek population.

## 4. Discussion

Various studies have examined the association between dental panoramic radiographs and BMD in adults and concluded that it is a good screening tool for diagnosing low bone density [6,12,19]. The existence of a similar correlation has not yet been established in the juvenile population. To the best of our knowledge, this is the first study that evaluated mandibular cortical bone quality and quantity using anthropometric indices in such detail and attempted to identify possible correlations with patient-related factors such as age and gender and factors directly associated with the occlusion distribution, as well as being the first to investigate the likelihood that bone density could be affected by factors such as the presence of deep caries lesions, missing teeth, extensive resin composites, and stainless-steel crowns.

According to the findings of this study, mean MCI values differed significantly between genders, with females having a more even and sharp endosteal edge of the cortex than boys. This is consistent with earlier research in the elderly and is possibly related to discrepant aging trajectories mediated by hormonal variations (e.g., growth hormone and oestrogen), body size, bone size, and geometry [20,21]. Studies in medically compromised children, though, failed to demonstrate such a correlation [8,22]. This could be attributed to the fact that our study sample included children up to 18 years of age, in whom the effects of hormonal changes and development have already begun to become more apparent.

In our study, a statistically significant increase in the prevalence of eroded bone in older patients, especially in patients 14 years of age and over, was observed. The age of 14 years appears to be the threshold path which there is a statistically significant difference that is slightly higher than that determined by the quantitative assessment, most likely because there were many boys in our sample whose puberty began late, and hence hormonal changes manifested at an older age. Furthermore, girls reach mandibular peak height velocity (PHV) around the age of 12, whereas boys reach this around the age of 14 years [23].

Similarly, regarding bone thickness, our results show a significant and linear increase from the ages of 8 to 9 years and onwards. Τhis was observed in all of the areas that were assessed (A, H-B, G, C-F, D-E, total) and can be attributed to the onset of puberty and bone maturation [24,25,26]. The relationship between skeletal development, facial growth, and mandibular growth, in particular, is debated. Numerous studies have indicated a strong correlation between the rate of facial growth, specifically the development of the mandible, and delayed or accelerated skeletal maturity, which is frequently accompanied by a corresponding deceleration or acceleration in facial growth [23,24]. Others, however, were unable to discover any conclusive links between skeletal maturity and mandibular growth during adolescence.

Low levels of oestrogen have been shown to affect craniofacial development, osteoporosis, osteoporotic fractures, osteoporosis in the alveolar bone, and abnormalities in the microarchitecture of the femur and mandible [27,28]. In a study utilizing a mouse model [29], it was hypothesized that one of the factors influencing the growth and development of the maxilla and mandible is oestrogen. Low oestrogen levels in women and teenage girls can be caused by a variety of disorders, and how they impact an individual depends on their age and general health. Clinicians should be aware of the potential effects of oestrogen shortage on the development of girls’ dental arches.

The results attained in the present study also demonstrate statistical differences in terms of the correlation between cortical bone consistency and type of dentition, namely mixed and permanent dentition. We concluded that in mixed dentition, an even and sharp endosteal margin of the cortex (C1) predominated, while this was less frequent in permanent dentition, and the frequency of cortical bone with an endosteal margin which shows semilunar defects increased (C2). This was expected because as age increases, changes in occlusion may alter the distribution and magnitude of forces applied to the mandible during chewing and biting. In general, growth of the craniofacial complex muscles enhances both chewing capacity and associated forces [30].

A non-statistically significant difference was shown for the correlation between cortical bone thickness and dental characteristics such as extensive caries, composites, missing teeth, and SSC. This is consistent with earlier research, which failed to demonstrate a positive connection between trabeculation of the jawbone and dental decay in children aged 8 to 13 years [31]. Another study in a paediatric population indicated a connection between extensive proximal caries and alveolar bone loss in primary dentition but on the grounds that it facilitates plaque retention [32].

Regarding missing teeth, our study did not find a correlation, which is not in accordance with previous studies in adults. When a tooth is lost, the underlying bone that once supported the tooth is no longer stimulated by the forces of chewing and biting. This lack of stimulation can lead to bone resorption, where the bone gradually diminishes in volume and density. The lack of a correlation in our study could be attributed to the participants’ age and the fact that the teeth had been missing for some time or that the presence of succedaneous teeth does not allow the mechanism of resorption to be activated. Regarding stainless-steel crowns, no correlation was recorded, a finding that is in agreement with the existing literature [26]. Studies have shown alveolar bone loss only in cases where crowns are judged radiographically as non-satisfactory [33,34,35].

Dentists, being the front-line members of the healthcare team, have the ability to identify paediatric patients at risk for bony defects through panoramic radiographs that are part of their routine dental examination. Given that more than 1.5 billion X-rays are performed annually in the world for dental purposes [36], dentist have the opportunity to use them not only for the detection of caries, etc., but also for the observation and evaluation of the bone of the jaw. Therefore, they might play a significant role in the early identification of individuals at risk for bone issues and serve as a source of early patient referral for care if bone alterations observed in panoramic radiographs could be equivalent to changes in QCT scans.

### Strengths and Limitations

This study’s strength was the large sample size of 660 X-rays from children and adolescents with a mean chronological age of 11.7 years, along with the outstanding intra- and inter-examiner reliability, which exceeded ≥0.8 in both cases.

However, as the current sample was recruited from the database of a specific institution that primarily consists of an urban population, the interpretation of the results may be limited to that specific population. Additionally, apart from the limitations in the design of retrospective studies, common limitations in linear measurements made on panoramic radiographs are mostly due to geometric distortion and unequal magnification. Nevertheless, the same machine was used, and this effect was minimal. Finally, the subjective evaluation of the dental panoramic radiographs could be considered as a source of potential reporting bias. However, given that the intra-examiner reliability was evaluated prior to the sample assessment, this was limited and barely significant.

More comprehensive and well-designed studies need to be conducted to confirm the correlations between age/gender and bone morphology or thickness. The information gathered by this research could be utilized as a control group in future investigations.

## 5. Conclusions

Within the limitations of this study, it can be concluded that gender and bone morphology according to the MCI are correlated in a statistically significant manner. The endosteal edge of the cortex is more consistent and acute in girls than in boys, with boys presenting semilunar abnormalities more frequently. Cortical bone thickness was significantly associated with the dentition developmental stage; i.e., cortical bone thickness was significantly higher in permanent dentition than in early or late mixed dentition. Bone quality and quantity do not seem to be significantly associated with any of the factors that can affect occlusion.

## Figures and Tables

**Table 1:** Distribution of mandibular cortex morphology according to MCI, by gender.

Stages	Right N (%)		Left N (%)		Total N (%)	
	Boys	Girls	Boys	Girls	Boys	Girls
C1	276 (84.7)	304 (91.0)	265 (81.3)	293 (87.7)	250 (76.9)	280 (84.1)
C2	49 (15.0)	30 (9.0)	60 (18.4)	41 (12.3)	74 (22.8)	53 (15.9)
C3	1 (0.3)	0 (0.0)	1 (0.3)	0 (0.0)	1 (0.3)	0 (0.0)
*p*-value ^1^	0.017		0.027		0.026	

C1: the endosteal margin of the cortex is even and sharp on both sides; C2: the endosteal margin shows semilunar defects (lacunar resorption) and/or seems to form endosteal cortical residues on one or both sides; C3: the cortical layer forms heavy endosteal cortical residues and is clearly porous; ^1^ Fisher’s exact test.

**Table 2:** Distribution of mandibular cortex morphology according to MCI, stratified by age group and gender.

**Boys**		**Age Groups**						
		**6–7 yrs**	**8–9 yrs**	**10–11 yrs**	**12–13 yrs**	**14–15 yrs**	**16–17 yrs**	**18 yrs**
		N (%)	N (%)	N (%)	N (%)	N (%)	N (%)	N (%)
**Stages**	C1	23 (63.9)	64 (90.1)	50 (92.6)	45 (77.8)	27 (55.1)	31 (70.5)	13 (76.5)
	C2	13 (36.1)	6 (8.5)	4 (7.4)	12 (22.2)	22 (44.9)	13 (29.5)	4 (23.5)
	C3	0 (0.0)	1 (1.4)	0 (0.0)	0 (0.0)	0 (0.0)	0 (0.0)	0 (0.0)
***p*-value ^1^** < 0.001								
**Girls**		**Age Groups**						
		**6–7 yrs**	**8–9 yrs**	**10–11 yrs**	**12–13 yrs**	**14–15 yrs**	**16–17 yrs**	**18 yrs**
		N (%)	N (%)	N (%)	N (%)	N (%)	N (%)	N (%)
**Stages**	**C1**	23 (71.0)	60 (84.5)	66 (85.7)	48 (80.0)	38 (92.7)	30 (88.2)	16 (84.2)
	**C2**	9 (29.0)	11 (15.5)	11 (14.3)	12 (20.0)	3 (7.3)	4 (11.8)	3 (15.8)
	**C3**	0 (0.0)	0 (0.0)	0 (0.0)	0 (0.0)	0 (0.0)	0 (0.0)	0 (0.0)
***p*-value ^1^** = 0.291								

^1^ Fisher’s exact test.

**Table 3:** The correlation between bone morphology and samples’ dental status.

	Bone Morphology *n* (%)			
Characteristics	C1	C2	Total	*p*-Value
**Caries**				
0	476 (80.5)	115 (19.5)	591	0.074 ^1^
1	18 (100.0)	0 (0.0)	18	
2	13 (81.3)	3 (18.8)	16	
3+	23 (71.9)	9 (28.1)	32	
**Composites**				
0	427 (82.0)	94 (18.0)	521	
1	32 (78.0)	9 (22.0)	41	
2	29 (74.4)	10 (25.6)	39	0.252 ^2^
3	18 (85.7)	3 (14.3)	21	
4+	24 (68.6)	11 (31.4)	35	
**SCC**				
0	503 (80.5)	122 (19.5)	625	0.915 ^1^
1	15 (88.2)	2 (11.8)	17	
2	8 (80.0)	2 (20.0)	10	
3	4 (80.0)	1 (20.0)	5	
**Missing teeth**				
0	477 (80.7)	114 (19.3)	591	
1	33 (89.2)	4 (10.8)	37	
2	15 (65.2)	8 (34.8)	23	0.194 ^1^
3	3 (75.0)	1 (25.0)	4	
4	2 (100.0)	0 (0.0)	2	
**Dentition**				
Early mixed	166 (83.4)	33 (16.6)	199	
Late mixed	130 (84.4)	24 (15.6)	154	0.082 ^2^
Permanent	234 (77.0)	70 (23.0)	304	

^1^ Fisher’s exact test; ^2^ chi-square test.

**Table 4:** Results for correlation between sample characteristics and total mandibular cortical bone thickness.

Characteristics	Total Cortical Bone Thickness Mean	*p*-Value ^1^
Caries		
0	3.6311	
1	3.0969	0.083
2	2.6996	
3+	3.0482	
Composites		
0	3.5458	
1	3.3291	
2	3.8376	0.791
3	3.7524	
4+	3.7178	
SCC		
0	3.5878	
1	2.7611	
2	3.7000	0.398
3	3.2364	
Missing teeth		
0	3.5944	
1	3.2060	
2	3.6282	0.575
3	3.0624	
4	1.9198	
Dentition		
Early mixed	3.2142	
Late mixed	3.4024	<0.001
Permanent	3.8778	
*Bonferroni*		
*Early–late mixed*		0.999
*Early mixed–permanent*		<0.001
*Late mixed–permanent*		0.049

^1^ One-way analysis of variance (ANOVA).

**Table 5:** Formulated reference chart for mandibular cortical bone quality according to MCI related to age and gender.

Bone Quality		C1		C2		C3	
Gender		Boys	Girls	Boys	Girls	Boys	Girls
Age							
**6 years**		76%	71%	24%	29%	0%	0%
**7 years**		65%	75%	35%	25%	0%	0%
**8 years**		59%	69%	41%	31%	0%	0%
**9 years**		84%	75%	16%	25%	0%	0%
**10 years**		82%	90%	18%	10%	0%	0%
**11 years**		88%	94%	8%	6%	4%	0%
**12 years**		91%	91%	9%	9%	0%	0%
**13 years**		86%	85%	14%	15%	0%	0%
**14 years**		88%	84%	12%	16%	0%	0%
**15 years**		77%	50%	23%	50%	0%	0%
**16 years**		76%	88%	24%	12%	0%	0%
**17 years**		65%	89%	35%	11%	0%	0%
**18 years**		94%	77%	6%	24%	0%	0%

**Table 6:** Formulated reference chart for mandibular cortical bone quantity (mean width in mm) related to age and gender.

Bone Quantity		Point A		Point B		Point C		Point D		Total	
Gender		Boys	Girls	Boys	Girls	Boys	Girls	Boys	Girls	Boys	Girls
Age											
**6 years**		2.02	2.16	3.49	4.04	3.62	4.13	3.84	4.40	3.42	3.91
**7 years**		3.51	2.38	4.20	3.20	4.29	3.18	4.84	3.49	4.31	3.16
**8 years**		3.22	2.84	4.16	2.84	4.51	3.93	4.78	4.42	3.13	2.00
**9 years**		3.16	2.82	3.84	3.51	4.13	3.58	4.60	4.24	2.27	2.02
**10 years**		2.93	2.76	3.96	3.51	4.09	3.53	4.44	3.87	2.20	1.96
**11 years**		3.04	2.84	3.58	3.53	3.58	3.44	3.82	3.64	2.00	1.93
**12 years**		3.20	2.60	3.91	3.33	3.87	3.36	4.13	3.58	2.27	1.84
**13 years**		2.82	2.42	3.44	3.20	3.62	3.00	4.02	3.16	2.00	1.69
**14 years**		2.78	2.20	3.40	2.80	3.53	2.73	3.51	2.96	1.89	1.53
**15 years**		2.36	3.58	2.91	4.51	2.84	4.58	2.78	4.71	1.56	2.49
**16 years**		3.18	2.56	4.11	3.47	4.16	3.71	4.27	3.87	2.24	1.96
**17 years**		2.36	3.20	3.07	4.64	3.16	4.76	3.18	4.82	1.69	2.49
**18 years**		3.87	3.62	5.31	4.58	5.11	4.62	5.20	4.76	2.78	2.76

## Data Availability

The data supporting this study’s findings are available on request from the corresponding author.
